# Intrauterine natural killer cell therapy for unexplained recurrent pregnancy failure

**DOI:** 10.1093/nsr/nwag332

**Published:** 2026-06-08

**Authors:** Hui Zhu, Xianghui Du, Yan Xu, Taishun Li, Biyun Xu, Yonggang Zhou, Fangting Lu, Xianhong Tong, Haixiang Sun, Haiming Wei, Yali Hu, Binqing Fu

**Affiliations:** Department of Obstetrics and Gynecology, Nanjing Drum Tower Hospital, Affiliated Hospital of Medical School, Nanjing University, China; State Key Laboratory of Immune Response and Immunotherapy, Department of Obstetrics and Gynecology, The First Affiliated Hospital of USTC, Center for Advanced Interdisciplinary Science & Biomedicine of IHM, Division of Life Sciences and Medicine, University of Science and Technology of China, China; Department of Obstetrics and Gynecology, Nanjing Drum Tower Hospital, Affiliated Hospital of Medical School, Nanjing University, China; Department of Obstetrics and Gynecology, Nanjing Drum Tower Hospital, Affiliated Hospital of Medical School, Nanjing University, China; Department of Obstetrics and Gynecology, Nanjing Drum Tower Hospital, Affiliated Hospital of Medical School, Nanjing University, China; State Key Laboratory of Immune Response and Immunotherapy, Department of Obstetrics and Gynecology, The First Affiliated Hospital of USTC, Center for Advanced Interdisciplinary Science & Biomedicine of IHM, Division of Life Sciences and Medicine, University of Science and Technology of China, China; State Key Laboratory of Immune Response and Immunotherapy, Department of Obstetrics and Gynecology, The First Affiliated Hospital of USTC, Center for Advanced Interdisciplinary Science & Biomedicine of IHM, Division of Life Sciences and Medicine, University of Science and Technology of China, China; State Key Laboratory of Immune Response and Immunotherapy, Department of Obstetrics and Gynecology, The First Affiliated Hospital of USTC, Center for Advanced Interdisciplinary Science & Biomedicine of IHM, Division of Life Sciences and Medicine, University of Science and Technology of China, China; Department of Obstetrics and Gynecology, Nanjing Drum Tower Hospital, Affiliated Hospital of Medical School, Nanjing University, China; State Key Laboratory of Immune Response and Immunotherapy, Department of Obstetrics and Gynecology, The First Affiliated Hospital of USTC, Center for Advanced Interdisciplinary Science & Biomedicine of IHM, Division of Life Sciences and Medicine, University of Science and Technology of China, China; Department of Obstetrics and Gynecology, Nanjing Drum Tower Hospital, Affiliated Hospital of Medical School, Nanjing University, China; State Key Laboratory of Immune Response and Immunotherapy, Department of Obstetrics and Gynecology, The First Affiliated Hospital of USTC, Center for Advanced Interdisciplinary Science & Biomedicine of IHM, Division of Life Sciences and Medicine, University of Science and Technology of China, China

Unexplained recurrent pregnancy loss (URPL) and unexplained recurrent implantation failure (RIF) represent significant clinical challenges in the field of human reproduction. Here, we report the promising pregnancy outcomes of 15 patients with URPL/RIF related to uterine natural killer (NK) cell abnormalities treated with induced CD49a^+^ decidual-like NK (idNK) cells from autologous peripheral blood (ChiCTR2100053633). Ongoing pregnancies and live births were achieved in 80% (12/15) of patients after two cycles of idNK cell therapy. Furthermore, no serious treatment-related adverse events were observed among all participants. These results indicate that further clinical studies assessing idNK cell treatment in URPL and RIF related to uterine NK cell disorders are warranted.

URPL was defined as having two or more consecutive miscarriages with no identifiable underlying cause [1]. RIF was defined in this study as the failure to achieve a clinical pregnancy after two or more embryo transfers (ETs) of good-quality embryos, with unknown causes. Previous findings from RIF cohorts and large randomized trials have shown that the cumulative live birth rates in such patients ranged from only 23.1% to 38.8% [2,3]. Similarly, the live birth rate of URPL patients who underwent frozen ET after cumulative *in vitro* fertilization (IVF) cycles was only ∼33.6% [4]. These data underscore the limited efficacy of current assisted reproductive strategies for these refractory conditions. Moreover, in unexplained infertility patients with normal ovulation but impaired implantation attempting unassisted conception, the sustained pregnancy rate and live birth rate were as low as ∼7% [5]. The modest success of IVF-ET and the profoundly low natural pregnancy rates highlight the need for novel therapies to improve endometrial receptivity, which are applicable to both assisted reproduction and natural conception.

Decidual NK (dNK) cells, the largest population of cells at the human maternal–fetal interface, possess a CD56^bright^CD16^–^ phenotype and express high levels of tissue-resident specific markers, including CD49a, CD103, and CD69. These NK cells exhibit unique functional capabilities, including the ability to promote decidualization, immune tolerance, trophoblast invasion, placental vascular growth, and fetal growth, via a noncytotoxic mechanism [6]. Accumulating evidence has demonstrated that an abnormal percentage or function of CD56^bright^ NK or tissue-resident NK (trNK) cells is closely associated with an impaired endometrial environment, resulting in miscarriage or fetal growth disorders [7]. Recently, we successfully performed noninvasive menstrual blood (MB) analysis to evaluate uterine endometrial immune cells [8]. Like dNK cells, more than 70% of

MBNK cells from healthy donors are CD56^bright^ NK cells, with a CD49a^+^ trNK cell phenotype. In contrast, compared with control cells, MBNK cells from patients with URPL have far fewer CD56^bright^ NK and CD49a^+^ trNK cells [8]. Using a non-feeder cell induction system, we also established a highly efficient, optimized differentiation protocol for the generation of idNK cells from autologous peripheral blood-derived NK cells, which are phenotypically and functionally similar to native human dNK cells with trNK markers and are highly capable of promoting both fetal growth and uterine artery blood flow [9]. These studies established the foundation for the clinical translation of idNK cells to human patients.

This single-arm clinical trial was approved by the Ethics Committee of Nanjing Drum Tower Hospital, Affiliated Hospital of Medical School, Nanjing University (2021-547-02) and registered at chictr.org.cn (registration number: ChiCTR2100053633). The primary outcomes of this study were the ongoing pregnancy rate, live birth rate and safety related to idNK cell treatment. Written informed consent was obtained from all patients after detailed explanations of the treatment procedure with potential benefits and risks were provided.

MB analyses were performed for 61 patients with URPL or RIF to evaluate their uterine NK cell status during the clinical trial enrollment period. MB samples were collected on the second day of each patient’s menstrual cycle via a menstrual cup, as previously reported [8]. Compared with the high proportion of NK cells with more than 70.0% CD56^bright^ and CD49a^+^ trNK subsets in normal MB donors, 51.16% of URPL (22/43), and 61.11% of RIF (11/18) patients had less than a 20% proportion of NK cells in CD45^+^ lymphocytes, accompanied by extremely low proportions of CD49a^+^ trNK cells and CD16^−^ and CD49a^+^CD16^−^ noncytotoxic NK cells (Fig. 1A and B). Patients who presented with an impaired functional uterine trNK cell profile in the MB (defined as a proportion of CD3^−^CD56^+^ NK cells in CD45^+^ lymphocytes <20% combined with a proportion of CD49a^+^ trNK cells <15% in CD45^+^ lymphocytes) and met the inclusion and exclusion criteria (details shown in the Supplementary Methods) were selected as candidates for idNK cell therapy.

**Figure 1.**
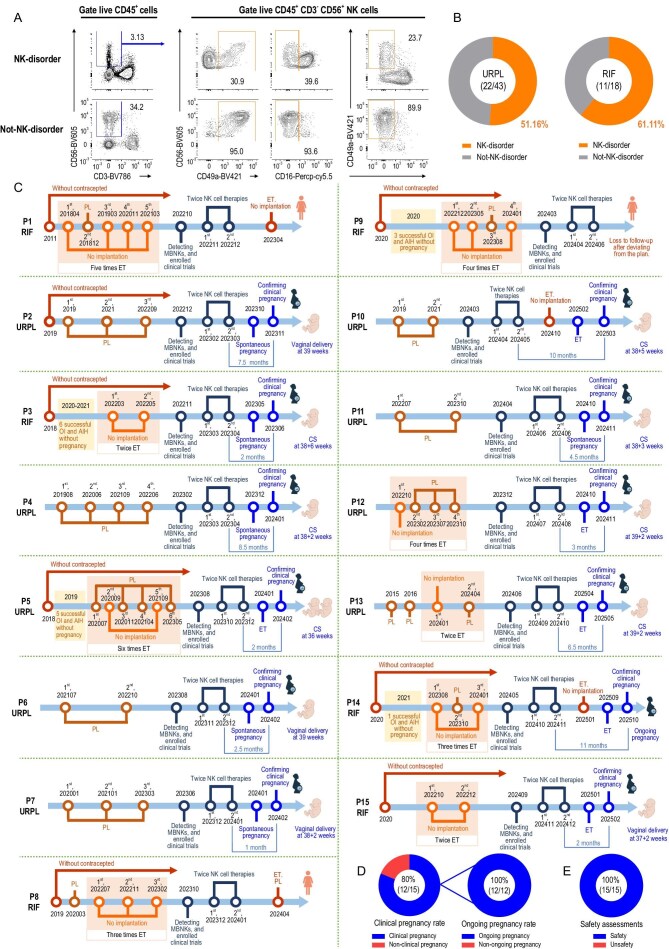
Autologous induced decidual-like natural killer (idNK) cell therapy improves pregnancy outcomes to 80% of URPL and RIF. (A) Flow cytometry was used to analyze the proportion of NK cells among CD45^+^ MB lymphocytes isolated from URPL and RIF patients. Lymphocytes were gated by SSC (side scatter) and FSC (forward scatter), and their flow cytometric profile with CD45^+^ immune cell staining was used to further analyze CD56, CD3, CD49a, and CD16 expression. (B) The proportions of uterine NK cell abnormalities in URPL (22/43) and RIF (11/18) patients. (C) Timeline showing patient medical history, the key time points of this study, and the pregnancy outcomes of the patients who participated in this clinical trial. (D) Clinical pregnancy and ongoing pregnancy rates achieved during the clinical trial. (E) Safety assessments of this clinical trial. AIH, artificial insemination by husband; CS, cesarean section; ET, embryo transfer; MBNKs, menstrual blood NK cells; OI, ovulation induction; and PL, pregnancy loss.

The process for screening, enrolling, and excluding patients in this clinical trial is presented in Fig. S1: fifteen patients were enrolled and completed idNK cell therapy in this trial, including 9 patients with URPL and 6 with RIF. The basic information of the participants is shown in Table S1. Among the 15 patients, 14 adhered to the study protocol. One patient (P9) violated the protocol by receiving an intrauterine infusion of autologous peripheral blood mononuclear cells (PBMCs) in an external clinical trial after idNK cell treatment and was subsequently lost to follow-up.

Eligible patient PBNK cells were isolated and then expanded *ex vivo* into CD49a^+^ idNK cells under stringent quality control (Fig. S2). Each patient received two intrauterine infusions of these autologous cells during two consecutive menstrual cycles and was advised to prepare for natural conception or IVF-ET during the next cycle. Once a pregnancy was confirmed, the follow-up assessments, including transvaginal ultrasonography at ∼40 days after the last menstrual period to confirm the presence of a gestational sac and embryo within the uterine cavity, nuchal translucency ultrasound assessment between 11 and 13^+6^ gestational weeks (GW), ultrasound screening for fetal structural anomalies between 20 and 24 GW, ultrasound scan for fetal growth evaluation at 28 GW, maternal pregnancy complications, gestational age at delivery, and neonatal birth weight, height, Apgar scores, and birth defects, were recorded.

Among the 15 patients (9 URPL and 6 RIF) who completed idNK cell therapy, 12 achieved ongoing pregnancies, including 6 spontaneous pregnancies and 6 conceptions via ET, and the median interval between completing the second idNK cell treatment and confirming a clinical pregnancy was 3.75 months (interquartile range 2.00–8.25 months). Ten of them (P2–P4, P6, P7, P10–P13, and P15) were singleton pregnancies that resulted in live births. One (P14) was an ongoing pregnancy, 37 GW, on 20 May 2026. The remaining patient (P5) had twins and underwent a cesarean section at 36 GW because of intrahepatic cholestasis (Fig. 1C). Two patients (P1 and P8) underwent ETs, but unfortunately, both experienced failed ET cycles. P9 received an intrauterine infusion of PBMCs after two idNK cell treatments and was then lost to follow-up (Fig. 1C). In summary, ongoing pregnancy and live birth were achieved in all 9 URPL patients (100%) and in 3 of the 6 RIF patients (50%). The 3 unsuccessful RIF cases included one case of implantation failure following ET, one case of biochemical pregnancy, and one case of loss to follow-up due to protocol violation. The overall ongoing pregnancy rate was 80% (12/15) (Fig. 1D).

Among the 12 patients who achieved pregnancy after idNK cell therapy, routine supportive medications (for example, anticoagulant therapy with heparin, aspirin or bromocriptine to control prolactin levels) were employed. Crucially, these pregnancies progressed to success, despite the use of similar regimens in previously failed cycles, highlighting idNK infusion as the decisive variable (Table S2).

The follow-up details for the 12 offspring are presented in Table S3. The birth weight and height of the neonates and infants were within the normal ranges. One case of ventricular septal defect occurred in an infant whose mother had both gestational diabetes mellitus (GDM) and intrahepatic cholestasis of pregnancy (ICP); the defect was surgically corrected at 4 months of age, with full recovery. The other case involved mild left ear dysplasia in an infant whose mother also had GDM; this infant currently has normal language development, suggesting a favorable prognosis. Both GDM and ICP are established risk factors associated with an increased incidence of congenital anomalies, including cardiac and auditory system defects. Therefore, while these events warrant careful consideration, the presence of these well-established obstetric confounders precludes a definitive causal attribution to idNK cell intervention.

From the perspective of safety assessment, we investigated body temperature, vaginal bleeding, lower abdominal pain, routine blood analysis, and C-reactive protein (CRP) levels on the second day after the procedure. Analysis revealed that all participants had normal temperatures, routine blood analysis results, and CRP levels and had no abnormal vaginal bleeding or lower abdominal pain (Fig. 1E and Table S4). Therefore, intrauterine idNK cell therapy demonstrated favorable safety in all participants.

Therefore, we report the clinical screening of uterine NK cell abnormalities related to URPL/RIF with non-invasive MB detection and then applied autologous CD49a^+^ idNK cells to conduct targeted treatment. The results revealed that of the 15 enrolled patients who completed idNK cell treatment, 12 (all 9 URPL patients and 3 of the 6 RIF patients) achieved clinically stable pregnancies, culminating in ongoing pregnancy and live birth during the follow-up period. Notably, half of the pregnant patients (6 of 12 patients) experienced spontaneous pregnancies following therapy. These findings indicate that the effect of idNK cell therapy may extend beyond supporting only ET, broadly enhancing endometrial receptivity and facilitating both natural and assisted conception.

URPL and RIF are often linked to immune dysfunction; however, no consistently effective immunomodulatory treatment has been established. This stems from the absence of validated diagnostic tests to confirm an immune-mediated etiology coupled with a lack of methodologies for appropriately selecting patients and objectively verifying therapeutic efficacy [10]. Moreover, the prevalent clinical practice of immunosuppression in URPL/RIF, which is often initiated on the basis of PBNK cell testing, is fundamentally flawed [10]. Widely used nonspecific immunosuppressive agents, such as glucocorticoids and intravenous immunoglobulin (IVIg), have not demonstrated consistent efficacy in improving ongoing pregnancy or live birth rates and carry potential risks of adverse effects [3]. Importantly, endometrial assessments in our cohort revealed compromised rather than hyperactive uterine NK-related functionality. Therefore, the therapeutic goal should shift from broad immunosuppression to targeted mucosal immune reconstitution. Our approach of intrauterine infusion of idNK cells embodies this paradigm shift, with the goal of restoring a receptive endometrial microenvironment by replenishing and modulating local NK cell function. The promising outcomes observed in this exploratory study provide preliminary clinical support for this conceptual advance.

Unlike the high-dose intravenous NK cell infusions (10⁹–10^10^ cells) used to treat malignant tumors, we employed a low-dose (10⁶ cells) direct intrauterine infusion of CD49a^+^ idNK cells, with the following key advantages. First, previous studies have suggested that the observed engraftment of adoptively transferred CD49a⁺ idNK cells is likely facilitated by their intrinsic tissue residency program, characterized by high CD49a expression, which mediates adhesion to collagen-rich endometrial tissue [7]. Second, the peri-implantation endometrial microenvironment, which is known to secrete specific chemokines [11], may actively recruit these cells. Third, it is easy and safe to deliver CD49a^+^ idNK cells into the uterine cavity. Therefore, the intrauterine perfusion strategy ensures targeted delivery, high local bioavailability and avoidance of systemic clearance.

This study has several limitations. First, as a single-arm pilot trial, the findings necessitate confirmation through large-scale, multicenter randomized controlled trials to definitively establish the efficacy and safety of idNK cell therapy. Second, we adopted a broader definition of RIF (including patients with only two prior failed ETs) to address the ethical and clinical imperative of offering a potential therapeutic option to patients with depleted embryo reserves and no established alternatives. This pragmatic approach justified earlier MBNK cell screening and, consequently, earlier idNK cell intervention. Third, while our data demonstrate that intrauterine CD49a⁺ idNK cells improve pregnancy outcomes, the precise molecular mechanisms through which these cells exert their effects remain to be elucidated.

Overall, our findings support further clinical studies in this direction and mark a step forward in the development of personalized idNK cell therapy to restore the endometrial mucosal immune environment and treat related reproductive disorders.

## Supplementary Material

nwag332_Supplemental_File

## RESOURCE AVAILABILITY

Further information and requests for resources should be directed to and will be fulfilled by the lead contact at fbq@ustc.edu.cn.
