# Supplementary material for: Intrauterine natural killer cell therapy for unexplained recurrent pregnancy failure
Source: Natl Sci Rev. 2026 Jun 8;13(13):nwag332. doi: 10.1093/nsr/nwag332 (PMC13335108; doi:10.1093/nsr/nwag332)
Supplement: nwag332_Supplemental_File [file nwag332_supplemental_file.docx]

**Supplementary Data**

**Intrauterine NK Cell Therapy for Unexplained Recurrent Pregnancy Failure**

Hui Zhu^1#^, Xianghui Du^2#^, Yan Xu^1^, Taishun Li^1^, Biyun Xu^1^, Yonggang Zhou^2^, Fangting Lu^2^, Xianhong Tong^2^, Haixiang Sun^1^, Haiming Wei^2^*, Yali Hu^1^*, Binqing Fu^2,3^*

**Affiliations:**

1. Department of Obstetrics and Gynecology, Nanjing Drum Tower Hospital, Affiliated Hospital of Medical School, Nanjing University, Nanjing 210008, China

2. State Key Laboratory of Immune Response and Immunotherapy, Department of Obstetrics and Gynecology, The First Affiliated Hospital of USTC, Center for Advanced Interdisciplinary Science & Biomedicine of IHM, Division of Life Sciences and Medicine, University of Science and Technology of China; Hefei, Anhui, 230001, China

3. Lead contact

# These authors contribute equally.

*Correspondence: fbq@ustc.edu.cn (B.F.); yalihu@nju.edu.cn (Y.H) or ustcwhm@ustc.edu.cn (H.W.)

**This document contains:**

**Supplementary Methods**

**Supplementary References**

**Supplementary Figures S1, S2**

**Supplementary Tables S1, S2, S3, S4**

**Supplementary Methods**

**Experimental model and study participant details**

This clinical study was conducted in accordance with the Declaration of Helsinki and approved by the Ethics Committee of Nanjing Drum Tower Hospital, Affiliated Hospital of Medical School, Nanjing University (Reference: 2021-547-02). The trial was registered with the Chinese Clinical Trial Registry (ChiCTR2100053633) prior to the start of patient recruitment. This report describes the 15 participants who were enrolled in this trial, with their clinical characteristics, follow-up data, and pregnancy outcomes detailed in **Supplementary Tables: Table S1, S2 and S3**. Human menstrual blood (MB) and peripheral blood (PB) samples were obtained from the patients who participated in this clinical trial. Each patient provided written informed consent.

Unexplained recurrent pregnancy loss (URPL) and unexplained recurrent implantation failure (RIF) patients with abnormal MB natural killer (NK) cell screening results were eligible if they met the following criteria: (1) women aged 22 to 40 years, with intention to conceive; (2) having normal ovarian function or frozen embryos; (3) endometrium thickness ≥ 7 mm, as measured by vaginal ultrasound before ovulation or at the mid-luteal phase; (4) 18 kg/m2 < body mass index (BMI) < 30 kg/m2; (5) having normal thyroid function. Patients were excluded if they had any of the following situations: (1) having the history of severe hypersensitivity to any types of drugs; (2) having progesterone receptor modulators (e.g., asoprisnil) or intrauterine infusion of cells that may impair NK cell survival or function; (3) either of the couple having an abnormal chromosome karyotype without normal embryos; (4) severe endometriosis or uterine fibroids affecting the shape of the uterine cavity, uterine malformation, or intrauterine adhesion; (5) severe vaginitis and endometritis; (6) other severe diseases or conditions that may potentially confound the assessment of pregnancy outcomes, including but not be limited to: severe coagulation dysfunction, abnormal liver or kidney function, uncontrolled hypertension, diabetes, uncontrolled autoimmune diseases, and malignant tumors.

**Menstrual blood collection**

Many efforts have been made to determine the numbers and cytotoxic potential of PBNK cells in infertile women to determine the status of uterine NK cells^1–3^. Unfortunately, PBNK cell testing results often reflect CD56^dim^ NK cells but not tissue-resident NK (trNK) cells. We previously successfully established non-invasive MB analysis to evaluate uterine endometrial immune cells^4^. This approach provides a fundamental method with which we can recognize URPL and RIF patients with abnormal uterine NK cells. MB samples were collected on the second day of each patient’s menstrual cycle. Approximately 3–5 hours later, the patients were squatted, and the menstrual cup was carefully removed to collect the MB. Each sample of MB was filtered through a 200-mesh iron screen into a centrifuge tube.

**Flow cytometry assays**

Cell suspensions were surface-labeled with human antibodies at 4°C for 30 min in the dark, washed twice with Phosphate Buffered Solution, and then tested. Following antibody staining, red blood cells were removed from the MB samples by lysis buffer (Beijing Tongshengshidai Biotechnology Co., LTD, Beijing, China). Flow cytometry was performed on a BD Celesta flow cytometer, and the data were analyzed by FlowJo software. All antibodies used for flow cytometry staining are listed in the key resources table.

**Generation of autologous induced decidual-like NK (idNK) cells**

On the second day of the menstrual cycle, we collected 40 mL of blood from the patient’s antecubital veins. Plasma was obtained by centrifuging the blood samples at 800 × g for 10 min followed by heat inactivation at 56°C for 30 min. Peripheral blood mononuclear cells (PBMCs) were isolated using Ficoll density gradients (TBD, Tianjin, China). PBNK cells were purified by negative selection with a magnetic-activated cell sorter (MACS) kit (Miltenyi Biotec, Germany). The idNK cells were cultured and identified according to our previously published work^5^. Efficacy of both mouse and human sources of idNK cells were evaluated and pregnancy promoting functionality was demonstrated in mouse models, indicating idNK cells had therapeutic potential for pregnancy complications^5–7^. The cells were cultured in a thousand-level purified laminar flow laboratory, which was subjected to annual environmental monitoring and inspected every two years by the National Health and Family Planning Commission of China, and is certified to perform cell culture work related to assisted reproduction.

The criteria for the use of idNK cells were a proportion of viable cells greater than 90%, a proportion of CD49a^+^ trNK cells greater than 80%, with high levels of CD9, CD39 and CD103 expression, and the percentage of CD16^+^ NK cells significantly decreased than PBNK. The cells were also confirmed to have low cytotoxicity towards K562 cells. Bacteria, fungi, mycoplasma and urea plasma were all negative, and endotoxin levels were <0.01 EU/mL. Manufacturing processes of idNK cells from the all patients enrolled in this study were subject to in-process quality control and release testing, as shown in **Supplementary Fig. S2**. These results demonstrated that the idNK cells generated from all patients met the final stage quality control criteria. These fresh patient-derived autologous idNK cells were kept in sodium chloride injection, injected into sterile cryopreservation tubes and transported to the clinical site within 4 hours at approximately 4°C.

**Cytotoxicity assays**

The human leukemic cell line, K562, was used as target cells, and was purchased from the Shanghai Cell Bank (Chinese Academy of Sciences, Shanghai, China). The cytotoxic activity of the NK cells was determined by carboxy fluoresce in succinimidylester (CFSE)-based cytotoxicity assays. The target cells were labeled with 5 µM CFSE (Invitrogen, USA) for 15 min at 37°C. NK cells (as effector cells) and K562 (as target cells) were then added to 96-well plates at a 10:1 effector-target ratio for 5 hours at 37°C and 5% CO_2_. Then, the cells were cultured in RPMI-1640 (VivaCell, Shanghai, China) containing 10% FBS (VivaCell, Shanghai, China). To detect dead cells, 7-AAD (BD Biosciences, USA) was added and the samples were directly analyzed by flow cytometry. The percentage of cytotoxicity was calculated using the proportion of 7-AAD^+^ cells was determined as follows: % cytotoxicity = 100 × (experimental group - average control group)/ (100 - average control group).

**Cell viability**

Cell viability was determined by gently mixing 1:1 Trypan Blue staining solution (2X, Beyotime Biotechnology, China) with a single cell suspension and stain for 3 min. Then, the cells were counted by Count Star. The cell survival rate was determined as follows: (total number of cells - number of blue cells)/ total number of cells ×100%.

**Intrauterine infusions of autologous idNK cells**

The patients were placed in the lithotomy position, the vulva, vagina and cervix were routinely disinfected with iodophor, and the cervix was exposed. Then, autologous idNK cells were slowly infused into the uterine cavity with a sterile Frydman catheter. On the second day after the infusion, the patient’s body temperature, vaginal bleeding, and lower abdominal pain were followed up. Blood routine and C-reactive protein (CRP) test results were analyzed. During the next menstruation cycle, the procedures were repeated.

**Quantification and statistical analysis**

Statistical significance was assessed utilizing GraphPad version 8. An unpaired two-tailed t-test was employed to evaluate statistical differences between the two groups. The data are presented as the mean ± standard error of the mean (SEM). For comparisons of data collected before and after treatment, paired t-tests were conducted. p < 0.05 was considered to indicate statistical significance.

**Key Resources Table**

| **REAGENT or RESOURCE** | **SOURCE** | **IDENTIFIER** |
| --- | --- | --- |
| **Antibodies** | | |
| BV421 Mouse Anti-Human CD49a | BD | Cat# 742357 |
| 647 Mouse Anti-Human CD16 | BD | Cat# 557710 |
| BV786 Mouse Anti-Human CD3 | BD | Cat# 563800 |
| Via-Probe Cell Viability Solution (7-AAD) | BD | Cat# 555816 |
| FITC anti-human CD39 Antibody | Biolegend | Cat# 328206 |
| PerCP/Cyanine5.5 anti-human CD3 | Biolegend | Cat# 300328 |
| PE/Cyanine7 anti-human CD49a Antibody | Biolegend | Cat# 328312 |
| PerCP/Cyanine5.5 anti-human CD16 | Biolegend | Cat# 302028 |
| Brilliant Violet 421 anti-human CD103 Antibody | Biolegend | Cat# 350214 |
| Brilliant Violet 510 anti-human CD45 Antibody | Biolegend | Cat# 368526 |
| Brilliant Violet 605 anti-human CD56 | Biolegend | Cat# 362538 |
| APC anti-human CD9 Antibody | Biolegend | Cat# 312108 |
| Zombie NIR™ Fixable Viability Kit | Biolegend | Cat# 423106 |
| **Chemicals, peptides, and recombinant proteins** | | |
| Animal-Free Recombinant Human IL-15 | PeproTech | Cat# AF-200-15 |
| Animal-Free Recombinant Human TGF-β1 | PeproTech | Cat# AF-100-21C |
| Chorionic gonadotropin for injection | LIVZON | Cat# H44020673 |
| **Experimental models: Cell lines** | | |
| K562 | Cell Bank, Chinese Academy of Sciences | TCHu191 |
| **Software and algorithms** | | |
| GraphPad Prism (v. 8.0.1) | GraphPad | https://www.graphpad.com |
| FlowJo_V10 | BD | https://www.flowjo.com |
| **Other** | | |
| Hemolytic agents for blood cell analysis | Beijing Tongsheng Shidai Biotechnology | Cat# Z6910001 |
| Sample density separation solution (endotoxin <0.12EU) | TBD | Cat# LTS10770125 |
| NK Cell Isolation Kit, human | Miltenyi Biotec | Cat# 130-092-657 |
| Serum-free Stem Cell Growth Medium | CellGenix | Cat# 20802 |
| Sodium Chloride Injection | Kelun Pharma | H10983065 |
| RPMI 1640 With L-Glutamine | VivaCell | Cat# C3010-0500 |
| Certified Fetal Bovine Serum (FBS) | VivaCell | Cat# C04001 |
| CFSE | Invitrogen | Cat# C34554 |
| Trypan Blue Staining Cell Viability Assay Kit | Beyotime Biotechnology | Cat# C0011 |

**Supplementary References**

1. Moffett, A. & Shreeve, N. First do no harm: uterine natural killer (NK) cells in assisted reproduction. *Hum Reprod* **30**, 1519–1525 (2015).

2. Sacks, G. Enough! Stop the arguments and get on with the science of natural killer cell testing. *Human Reproduction* **30**, 1526–1531 (2015).

3. Rai, R., Sacks, G. & Trew, G. Natural killer cells and reproductive failure—theory, practice and prejudice. *Human Reproduction* **20**, 1123–1126 (2005).

4. Tong, X. *et al.* Analysis of uterine CD49a+ NK cell subsets in menstrual blood reflects endometrial status and association with recurrent spontaneous abortion. *Cell Mol Immunol* **18**, 1838–1840 (2021).

5. Du, X. *et al.* Human-Induced CD49a+ NK Cells Promote Fetal Growth. *Front Immunol* **13**, 821542 (2022).

6. Fu, B. *et al.* Natural Killer Cells Promote Fetal Development through the Secretion of Growth-Promoting Factors. *Immunity* **47**, 1100-1113.e6 (2017).

7. Ni, X. *et al.* Cytokine-Based Generation of CD49a+Eomes−/+ Natural Killer Cell Subsets. *Front. Immunol.* **0**, (2018).

**Supplementary Figures**

**
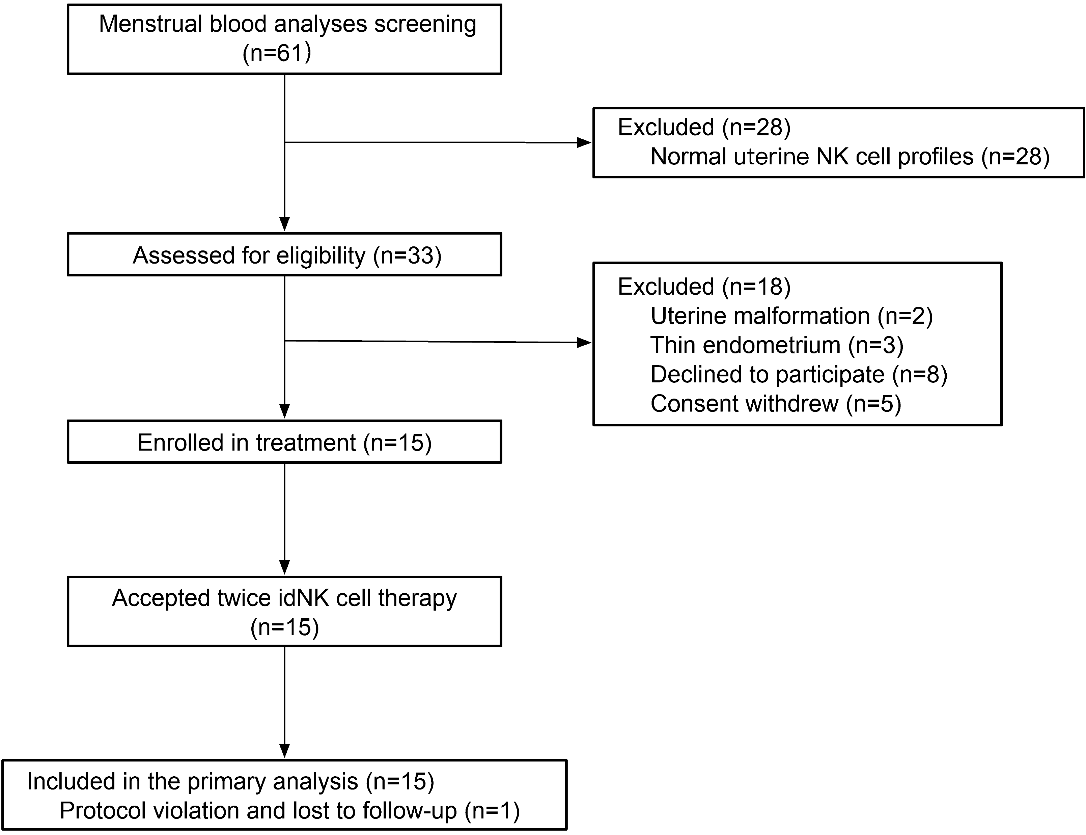
**

**Figure S1. Flow chart.** idNK, induced decidual-like natural killer.


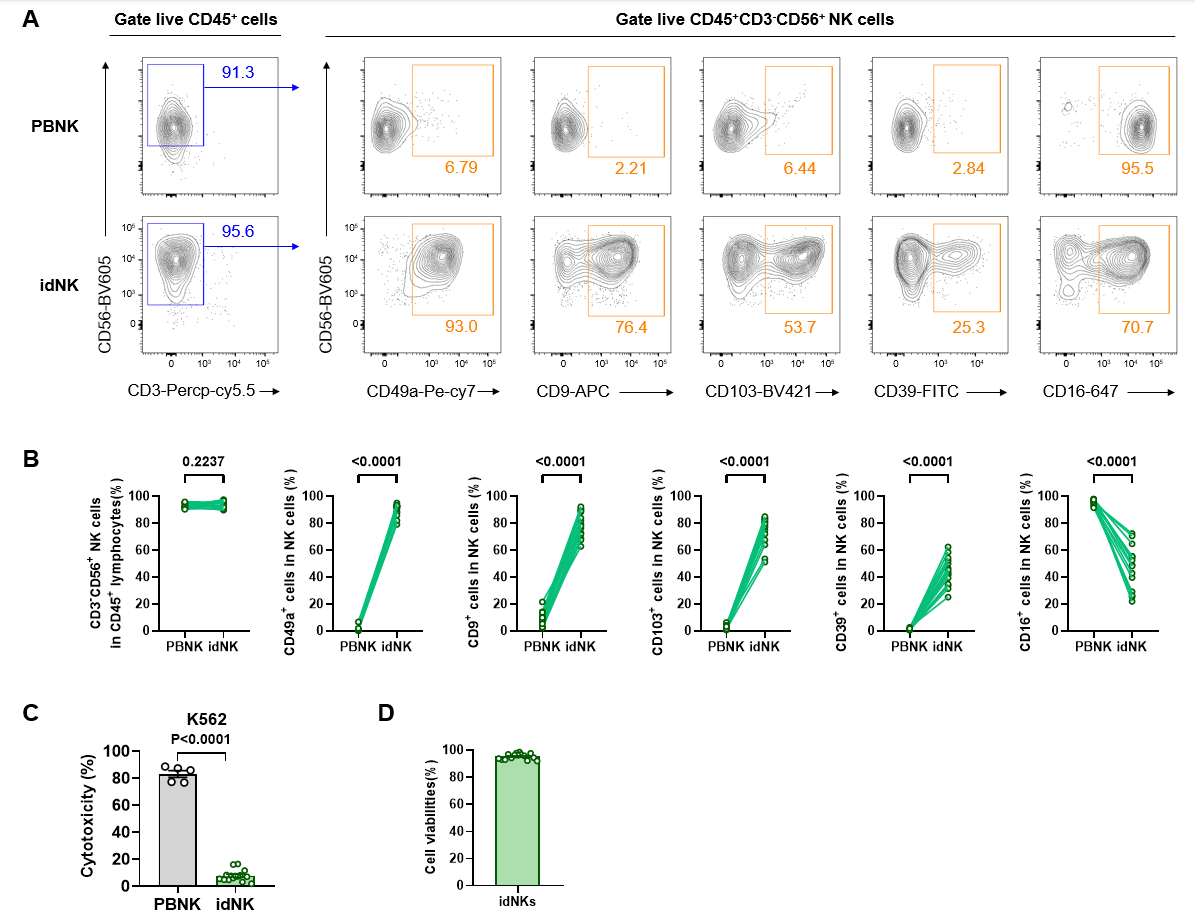


**Figure S2. Autologous induced decidual-like natural killer (idNK) cells from patients exhibit phenotype signatures similar to decidual NK (dNK) cells and have low cytotoxic capability. A and B.** The percentage of NK cells that expressed dNK-phenotypic markers before induction (Peripheral Blood NK, PBNK) and after induction (idNK) was determined by flow cytometry. The numbers in (A) show the percentage of each cell subset. Representative density plots (A) and statistical calculation of all samples (B). **C.** The direct cytotoxicity of idNK cells toward K562, as determined by flow cytometry. NK cells were used at a 10:1 effector to target ratio and incubated for 5 hours with K562 cells. The percentage of cytotoxicity was calculated used the proportion of 7-AAD^+^ cells as follows: % cytotoxicity = 100 × (experimental group - control group)/ (100 - control group). **D.** Cell viability of idNK cells before perfusion was assayed by trypan blue staining. Data represent mean ± SEM. Each data point represents an independent human sample, and n=15. p < 0.05 was considered statistically significant.

**Supplementary Tables**

**Table S1. Clinical characteristics of patients.**

| **Patient No.** | **Age of the cell therapy (years)** | **BMI (kg/m^2^)** | **AMH value before treatment (ng/mL)** | **Past obstetric history** | **Indication** | **Age of oocyte retrieval for ET post-therapy (years)** |
| --- | --- | --- | --- | --- | --- | --- |
| **P1** | 38 | 22.2 | 4.2 | 5 failed ET cycles (including 1 PL) | RIF | 36 |
| **P2** | 33 | 21.3 | 4.5 | 3 PL | URPL | NA |
| **P3** | 35 | 22.9 | 3.5 | 6 successful OI and AIH without pregnancy, 2 failed ET cycles | RIF | NA |
| **P4** | 30 | 21.6 | 4.6 | 4 PL | URPL | NA |
| **P5** | 30 | 22.3 | 6.8 | 5 successful OI and AIH without pregnancy, 6 failed ET cycles (including 4 PL) | URPL | 29 |
| **P6** | 28 | 23.2 | 3.2 | 2 PL | URPL | NA |
| **P7** | 33 | 23.7 | 4.8 | 3 PL | URPL | NA |
| **P8** | 40 | 21.6 | 3.8 | 1 PL, 3 failed ET cycles | RIF | 38 |
| **P9** | 31 | 25.0 | 2.0 | 3 successful OI and AIH without pregnancy, 4 failed ET cycles (including 1 PL) | RIF | 30 |
| **P10** | 35 | 20.1 | 2.4 | 2 PL | URPL | 35 |
| **P11** | 38 | 21.5 | 3.1 | 2 PL | URPL | NA |
| **P12** | 30 | 20.9 | 2.9 | 4 failed ET cycles (including 3 PL) | URPL | 28 |
| **P13** | 30 | 21.6 | 4.1 | 2 PL, 2 failed ET cycles (including 1 PL) | URPL | 29 |
| **P14** | 37 | 18.7 | 3.5 | 1 successful OI and AIH without pregnancy, 3 failed ET cycles (including 1 PL) | RIF | 36 and 38 |
| **P15** | 34 | 20.8 | 2.9 | 2 failed ET cycles | RIF | 34 |

**AIH, artificial insemination by husband; AMH, anti-Müllerian hormone; BMI, body mass index; ET, embryo transfer; NA, not applicable; OI, ovulation induction; P, patient; PL, pregnancy loss; RIF, recurrent implantation failure; URPL, unexplained recurrent pregnancy loss.**

**Table S2. Pregnancy complications and medication use in patients with successful pregnancy after idNK cell therapy.**

| **Patient No.** | **Previous medical history** | **Medication use in prior failed pregnancy attempts** | **Mode of pregnancy** | | **Medication use in pregnancy after idNK cell therapy** | **Maternal pregnancy complications** | **Ultrasound screening for fetal structural anomalies** | **Pregnancy Outcome** |
| --- | --- | --- | --- | --- | --- | --- | --- | --- |
| **P2** | Thrombophilia | Aspirin, heparin | SP | Aspirin, heparin | | No | No | Vaginal delivery at 39 weeks |
| **P3** | No | Progesterone, prednisone, aspirin | SP | Progesterone | | No | No | CS at 38+6 weeks |
| **P4** | No | Progesterone, aspirin, heparin | SP | Progesterone, aspirin, heparin | | NOAPS | No | CS at 38+2 weeks |
| **P5** | No | Progesterone, heparin, autologous PRP | ET | Progesterone, HCG, heparin, ursodeoxycholic acid | | ICP and GDM | No | Twin pregnancy and CS at 36 weeks |
|  |  |  |  |  |  |  | VSD |  |
| **P6** | Hyperprolactinemia | Bromocriptine mesilate; progesterone | SP | Bromocriptine mesilate; progesterone | | GDM | No | Vaginal delivery at 39 weeks |
| **P7** | Hypothyroidism | Levothyroxine sodium | SP | Levothyroxine sodium | | No | No | Vaginal delivery at 38+2 weeks |
| **P10** | No | Progesterone | ET | Progesterone | | No | No | CS at 38+5 weeks |
| **P11** | APS | Aspirin, heparin | SP | Aspirin, heparin, hydroxychloroquine | | GDM | VSD | CS at 38+3 weeks |
| **P12** | No | Progesterone, HCG, heparin | ET | Progesterone, HCG, heparin | | GDM | No | CS at 39+2 weeks |
| **P13** | No | Progesterone, aspirin, HCG | ET | Progesterone, aspirin | | No | No | CS at 39+2 weeks |
| **P14** | No | Progesterone, HCG | ET | Progesterone | | No | No | Ongoing pregnancy |
| **P15** | No | Progesterone | ET | Progesterone | | No | No | Vaginal delivery at 37+2 weeks |

**APS, antiphospholipid syndrome; CS, cesarean section; ET, embryo transfer; GDM, gestational diabetes mellitus; HCG, human chorionic gonadotropin; ICP, intrahepatic cholestasis of pregnancy; idNK, induced decidual-like natural killer; NA, not applicable; NOAPS: non-criteria obstetric antiphospholipid syndrome; P, patient;** **PRP: platelet-rich plasma; SP, spontaneous pregnancy; VSD, ventricular septal defect.**

| **Patient No.** | **Neonatal birth weight (g)** | **Neonatal birth height (cm)** | **Neonatal sex** | **Apgar score at 1/5 minute of newborns** | **Infant follow-up (period)^a^** | **Infant weight at last visit**  **(g)** | **Infant height at last visit (cm)** | **Infant head circumference at last visit**  **(cm)** | **Infant motor and cognitive ability at last visit** |
| --- | --- | --- | --- | --- | --- | --- | --- | --- | --- |
| **P2** | 3385 | 50 | Female | 9-10 | Healthy (12 months) | 10140 | 78 | 46 | Normal |
| **P3** | 3400 | 50 | Female | 10-10 | Healthy (12 months) | 10800 | 78 | 45 | Normal |
| **P4** | 3650 | 51 | Female | 8-9 | Healthy (12 months) | 9800 | 75 | 46 | Normal |
| **P5** | 2500 | 47 | Male | 10-10 | Healthy (12 months) | 9500 | 73 | 45 | Normal |
|  | 2150 | 43 | Female | 9-10 | VSD and right thumb with multiple fingers at birth; cardiac surgery peformed at 4 months of age and no abnormality in echocardiography at 12 months of age (12 months) | 9300 | 71 | 44 | Normal |
| **P6** | 3055 | 49 | Female | 9-10 | Left auricle malformation with left hearing loss, and right auricle and ear hearing normal at birth; language development normal at 12 months of age (12 months) | 10700 | 73 | 47 | Normal |
| **P7** | 3050 | 48 | Female | 9-10 | Healthy (12 months) | 9300 | 74 | 46 | Normal |
| **P10** | 3410 | 50 | Female | 10-10 | Healthy (1 month) | 4100 | 53 | 38 | Normal |
| **P11** | 2760 | 47 | Male | 10-10 | No obvious VSD in echocardiography at 2 months of age (6 months) | 6200 | 65 | 41 | Normal |
| **P12** | 3090 | 48 | Female | 10-10 | Healthy (6 months) | 8300 | 64.5 | 43 | Normal |
| **P13** | 3400 | 50 | Male | 9-10 | Healthy (1 month) | 3950 | 53 | 37 | Normal |
| **P14** | NA | NA | NA | NA | NA | NA | NA | NA | NA |
| **P15** | 2800 | 47 | Male | 10-10 | Healthy (3 months) | 6400 | 60 | 41.2 | Normal |

**Table S3. Details of follow-up in neonatals and infants.**

**cm, centimeter; g, gram; NA, not applicable; P, patient; VSD, ventricular septal defect.**

**^a^Infant follow-up period: defined as the interval between the neonatal birth and the last visit.**

**Table S4 | Safety assessments in patients after intrauterine infusion of idNK cells.**

| **Patient No.** | **Fever** | **Abnormal vaginal bleeding** | **Lower abdominal pain** | **Blood routine test and C-reactive protein test** |
| --- | --- | --- | --- | --- |
| **P1** | No | No | No | Normal |
| **P2** | No | No | No | Normal |
| **P3** | No | No | No | Normal |
| **P4** | No | No | No | Normal |
| **P5** | No | No | No | Normal |
| **P6** | No | No | No | Normal |
| **P7** | No | No | No | Normal |
| **P8** | No | No | No | Normal |
| **P9** | No | No | No | Normal |
| **P10** | No | No | No | Normal |
| **P11** | No | No | No | Normal |
| **P12** | No | No | No | Normal |
| **P13** | No | No | No | Normal |
| **P14** | No | No | No | Normal |
| **P15** | No | No | No | Normal |

**P, patient; idNK, induced decidual-like natural killer.**
